# Omega-3 polyunsaturated fatty acids improve intestinal barrier integrity—albeit to a lesser degree than short-chain fatty acids: an exploratory analysis of the randomized controlled LIBRE trial

**DOI:** 10.1007/s00394-023-03172-2

**Published:** 2023-06-15

**Authors:** Benjamin Seethaler, Katja Lehnert, Maryam Yahiaoui-Doktor, Maryam Basrai, Walter Vetter, Marion Kiechle, Stephan C. Bischoff

**Affiliations:** 1 Institute of Nutritional Medicine, University of Hohenheim, Fruwirthstr. 12, 70593 Stuttgart, Germany; 2 Institute of Food Chemistry, University of Hohenheim, Stuttgart, Germany; 3 Institute for Medical Informatics, Statistics and Epidemiology (IMISE), University of Leipzig, Leipzig, Germany; 4 Department of Gynecology, Center for Hereditary Breast and Ovarian Cancer, Klinikum Rechts der Isar, Technical University Munich and Comprehensive Cancer Center Munich, Munich, Germany

**Keywords:** Mediterranean diet, Omega 3 fatty acids, PUFAs, Gut barrier, Intestinal barrier, Gut permeability

## Abstract

**Abstract:**

**Purpose:**

Adherence to the Mediterranean diet is associated with beneficial health effects, including gastrointestinal disorders. Preclinical studies suggest that omega-3 polyunsaturated fatty acids (*n*-3 PUFAs), found in Mediterranean foods like nuts and fish, improve intestinal barrier integrity. Here, we assessed possible effects of *n*-3 PUFAs on barrier integrity in a randomized controlled trial.

**Methods:**

We studied 68 women from the open-label LIBRE trial (clinicaltrials.gov: NCT02087592) who followed either a Mediterranean diet (intervention group, IG) or a standard diet (control group, CG). Study visits comprised baseline, month 3, and month 12. Barrier integrity was assessed by plasma lipopolysaccharide binding protein (LBP) and fecal zonulin; fatty acids by gas chromatography with mass spectrometry. Median and interquartile ranges are shown.

**Results:**

Adherence to the Mediterranean diet increased the proportion of the *n*-3 docosahexaenoic acid (DHA) (IG + 1.5% [0.9;2.5, *p* < 0.001]/ + 0.3% [− 0.1;0.9, *p* < 0.050] after 3/12 months; CG + 0.9% [0.5;1.6, *p* < 0.001]/ ± 0%) and decreased plasma LBP (IG − 0.3 µg/ml [− 0.6;0.1, *p* < 0.010]/ − 0.3 µg/ml [− 1.1; − 0.1, *p* < 0.001]; CG − 0.2 µg/ml [− 0.8; − 0.1, *p* < 0.001]/ ± 0 µg/ml) and fecal zonulin levels (IG − 76 ng/mg [− 164; − 12, *p* < 0.010]/ − 74 ng/mg [− 197;15, *p* < 0.001]; CG − 59 ng/mg [− 186;15, *p* < 0.050]/ + 10 ng/mg [− 117;24, *p* > 0.050]). Plasma DHA and LBP (*R*^2^: 0.14–0.42; all *p* < 0.070), as well as plasma DHA and fecal zonulin (*R*^2^: 0.18–0.48; all *p* < 0.050) were found to be inversely associated in bi- and multivariate analyses. Further multivariate analyses showed that the effect of DHA on barrier integrity was less pronounced than the effect of fecal short-chain fatty acids on barrier integrity.

**Conclusions:**

Our data show that *n*-3 PUFAs can improve intestinal barrier integrity.

**Trial registration number**: The trial was registered prospectively at ClinicalTrials.gov (reference: NCT02087592).

**Supplementary Information:**

The online version contains supplementary material available at 10.1007/s00394-023-03172-2.

## Introduction

The intestinal barrier is a complex anatomical structure, protecting the host against gut microbes, food antigens, and toxins present in the gastrointestinal tract. A functioning intestinal barrier is required for gut health, whereas intestinal barrier impairment has been associated with numerous diseases such as cardiovascular disease, cancer, type 2 diabetes, and inflammatory bowel disease [1, 2].

Intestinal barrier function is affected by several endogenous and exogenous factors including diet, stress, excessive body weight, and low or extreme physical activity [1, 3, 4]. Previous findings have determined that the intestinal barrier plays a central role in disease occurrence, yet the mechanisms by which the barrier function is regulated are not well known. Dietary factors might play an important role here. We and others have shown that short-chain fatty acids, derived from bacterial fermentation of dietary fibers, improve intestinal barrier function [5–7]. Also, vitamins, minerals, amino acids and polyphenols might have an effect [8, 9]. Furthermore, several preclinical studies suggest that diet-derived omega-3 polyunsaturated fatty acids (*n*-3 PUFAs) improve intestinal barrier integrity [10, 11] yet to date there is no data including human subjects regarding this.

The Mediterranean diet describes the traditional dietary pattern in south Italy and Greece in the mid 1950s, which was characterized by a high intake of *n*-3 PUFAs like alpha-linolenic acid and docosahexaenoic acid (DHA) due to a regular consumption of seafood and nuts [12]. Such *n*-3 PUFAs have been shown to be cardioprotective mainly due to beneficial effects on atherosclerosis, arrhythmias, inflammation, and thrombosis [13]. Furthermore, there is evidence that they improve endothelial function, lower blood pressure, and significantly lower triglycerides [13]. Adherence to the Mediterranean diet has been associated with primary and secondary prevention of diseases, which are also linked to intestinal barrier impairment, including cardiovascular disease [14, 15], cancer [16–18], and type 2 diabetes [19]. Also, this diet has been shown to be effective in the prevention of and improvement of gastrointestinal disorders, including intestinal barrier impairment [5, 20].

In the present study, we aimed to assess possible associations between the Mediterranean diet, plasma fatty acid composition, and intestinal barrier integrity for the first time in a human study. We hypothesized that *n*-3 PUFAs improve intestinal barrier function, assessed by the two validated biomarkers plasma lipopolysaccharide binding protein (LBP) and fecal zonulin [21].

## Materials and methods

### Study design

Data for the present exploratory study derived from the randomized controlled LIBRE (Lifestyle Intervention Study in Women with Hereditary Breast and Ovarian Cancer) trial. The LIBRE study was a randomized (1:1 ratio), prospective, open-label, two-armed controlled multicenter trial, conducted in Germany. It aimed to test the effect of a structured lifestyle intervention program focussing on the Mediterranean diet and increased physical activity on cancer-relevant outcomes. The study included women at high risk for breast and ovarian cancer due to a pathogenic germline mutation in the BRCA1 and/or BRCA2 genes. BRCA mutations have been shown to be associated with an altered intestinal barrier function [5, 20] and it is suggested that intestinal barrier impairment is linked to breast cancer initiation and progression [22].

In the present explorative analyses we included all 68 participants from the completed LIBRE-1 study [23], which started in 2014. The LIBRE-1 study was a feasibility study with the number of participants who successfully completed the first 3 months of lifestyle intervention used as primary endpoint. A rate of 70% adherence or more was considered as success. Secondary endpoints comprised body mass index (BMI), physical activity, which is measured objectively by spiroergometry and is expressed as the ventilatory threshold (VT1), the analyses of omega fatty acids, and fecal metabolites. VT1 is an objective marker of physical fitness and represents the level of physical activity at which blood lactate accumulates faster than it can be cleared in spiroergometry. The sample size in LIBRE-1 was adjusted to this goal but was not calculated based on statistical assumptions and tests [23]. The main focus of the present analysis was to assess possible changes in the plasma fatty acid composition, especially in the proportion of n-3 PUFAs upon intervention. Considering the mean increase in the proportion of *n*-3 PUFAs in the plasma fatty acids between baseline and month 3, a post-hoc power calculation showed a power of 77% given an alpha error of 5% (intervention group 1.8% ± 2.3% [mean ± SD]; control group 0.3% ± 2.3%). The LIBRE-2 confirmatory study, which aims to include 600 women, started 2015 and recruitment is ongoing [24].

In LIBRE, women with a history of breast cancer prior to study start as well as women without previous breast cancer were included. Inclusion criteria were female sex, age between 18 and 69 years, a pathogenic BRCA1/2 mutation and written informed consent. Exclusion criteria comprised, among others, a BMI below 15 kg/m^2^, neoplastic diseases currently in treatment, as well as food allergies and/or dietary patterns which prevent the implementation of the Mediterranean diet, like veganism [23].

Individuals from the intervention group (*n* = 33) received a structured lifestyle-intervention program, consisting of a three-month intensive phase with bi-weekly group classes on the Mediterranean diet as well as professionally guided sport training, focussed on endurance-oriented exercises. The intensive phase was followed by a nine-month less intensive phase with monthly meetings. The control group (*n* = 35) were lectured once on the dietary recommendations of the German Nutrition Society (DGE) and once on the beneficial effects of regular physical activity on breast cancer incidence, prognosis, and recurrence at the beginning of the study. Study visits were at baseline, as well as 3 months (time point V1) and 12 months (time point V2) after baseline. Details on the enrolment, randomization, drop-outs, and available data for each time point are shown in the CONSORT flow chart in Supplementary Fig. 1.

The ethics review board of the Klinikum Rechts der Isar of the Technical University of Munich approved the study protocol (reference 5686/13) which was in accordance with the ethical standards laid down in the 1964 Declaration of Helsinki and its later amendments. The trial was registered at ClinicalTrials.gov (reference: NCT02087592).

### Dietary measurements

Two validated questionnaires were used to assess dietary habits. The Mediterranean Diet Adherence Screener (MEDAS), developed in the Prevención con Dieta Mediterránea (PREDIMED) studies [25], is a validated tool to measure adherence to the Mediterranean diet. As part of the LIBRE-1 study, we translated the original version into German and validated the German version again [26]. The MEDAS consists of 14 dichotomous questions, focussing on food consumption and typically Mediterranean dietary habits. Each question is scored with either 0 or 1, with 1 representing the answers that are related to the Mediterranean diet. Therefore, the MEDAS-Score ranges from 0 to 14, with 14 points representing the highest adherence to the Mediterranean diet. Two MEDAS questions imply meat consumption (#5 & #13, [26]) and were occasionally left out by vegetarians. Therefore, we calculated the MEDAS-Score as the percentage of the achieved score related to the achievable score (e.g. 7/14 = 50%; 7/13 = 54%).

In addition to the MEDAS, participants were asked to complete a 33-page long semi-quantitative Food Frequency Questionnaire (FFQ) established and validated by the European Prospective Investigation into Cancer and Nutrition (EPIC) consortium [27]. The EPIC-FFQ contains various questions asking qualitatively and quantitatively about food and beverage consumption, covering the previous 12 months (baseline), the previous 3-month-intervention phase (V1), or the previous 9-month-intervention phase (V2). Data input and data evaluation were performed using the study management system for health research, which has been developed by the Department of Epidemiology of the German Institute of Human Nutrition. The EPIC-FFQ provides the daily intakes of food groups (e.g. fruits, vegetables, nuts) and nutrients (e.g. fats, carbohydrates, protein). In the EPIC-FFQ results, *vegetable oil* refers to the sum of all vegetable oils consumed; *processed meat* refers to meat and meat products which have been processed by e.g. salting, curing, fermentation, smoking, and/or the addition of chemical preservatives (including e.g. bacon, ham, and sausages); *red meat* refers to unprocessed meat of red color, e.g. beef and pork.

Data from the EPIC-FFQ were adjusted for energy intake in accordance to Willett et al. [28]. Since the EPIC-FFQ does not per se measure adherence to the Mediterranean diet, we calculated the Mediterranean Diet Score (MedD-Score), a commonly used score established by Trichopoulou et al. [29]. Thus, in the present analyses, we included two independent scores which determine adherence to the Mediterranean diet, i.e. the MEDAS-Score and the MedD-Score. Dietary data for all variables and all time points is shown in Supplementary Table 1.

### Blood and fecal sample collection

Blood and fecal samples were collected in 2014 and 2015 at the participating study centers and sent to the University of Hohenheim overnight. All samples were stored at −80 °C and were analyzed in 2017 and 2018.

### Plasma fatty acid composition

Plasma fatty acids were transferred into fatty acid methyl esters (FAME) and were determined by gas chromatography with mass spectrometry (GC/MS). Afterwards, the proportion (%) of each fatty acid in the total fatty acid composition (= 100%) was determined. Therefore, each fatty acid is shown as the proportion of the respective fatty acid (%), as shown in Supplementary Table 2.

In detail, blood samples were collected in ethylenediaminetetraacetic acid (EDTA)-coated tubes. To separate the plasma, the samples were centrifuged at 500*g* for 7.5 min at 15 °C. Plasma fatty acid composition was assessed similar to a method used for fatty acid analysis in erythrocyte membranes, which was described previously [30]. In brief, 0.05 ml of plasma was supplemented with a solution containing 2 µg the internal standard 10,11-dichloro-undecanoic acid (DC 11:0) which was synthesized according to Thurnhofer and Vetter [31]. Transesterification (60 min at 80 °C) was carried out by adding 2 ml methanol with 1% sulphuric acid according to Wendlinger et al. [32]. Finally, 2.5 μg of the second internal standard, myristic acid ethyl ester (14:0 EE), was added to the resulting solution with the fatty acid methyl esters. This internal standard, which does not interfere with the fatty acid methyl esters in the samples, was used to level off variations in the instrumental performance between the individual measurements [33]. The final sample solutions were analyzed by GC/MS on a 5890 series II/5972A system (Hewlett-Packard, Waldbronn, Germany) equipped with a 60 m × 0.25 mm i.d. capillary column coated with 0.1 µm film thickness 10% cyanopropylphenyl, 90% bis-cyanopropyl polysiloxane (Rtx 2330, Restek, Belafonte, PA, USA) operated in selected ion monitoring (SIM) mode according to Thurnhofer et al. [34].

### Intestinal barrier biomarkers

All intestinal barrier biomarkers were analyzed using enzyme-linked immunosorbent assays following the manufacturer’s protocols. Zonulin was measured in fecal samples (K5600; Immundiagnostik AG, Bensheim, Germany). LBP was measured in plasma (REFs: DY870-05 and DY008; Bio-Techne GmbH, Wiesbaden, Germany).

### Short-chain fatty acids

To analyze fecal short-chain fatty acid (SCFA) levels, 400–500 mg feces were first diluted 1:4 in water. Then, 0.1 ml of 50% ortho-phosphoric acid (AppliChem GmbH, Darmstadt, Germany) was added before the samples were homogenized and filtered using polyester syringe filters (REF: 729033; Macherey–Nagel GmbH & Co. KG, Düren, Germany). Afterwards, 1 µl filtrate was analyzed using a capillary gas chromatograph (HP6890 Series; Hewlett Packard Corp., Paolo Alto, California, USA) with a flame ionization detector using the column OPTIMA-FFAP (REF: 726344.10; Macherey–Nagel GmbH & Co. KG, Düren, Germany) with standards for all SCFAs (Merck Schuchardt OHG, Hohenbrunn, Germany). Fecal dry mass was assessed by drying 300–500 mg feces overnight at 103 °C [35]. The SCFA data are expressed in relation to dry mass to overcome bias due to differing fecal water contents.

### Statistical analyses

Prior to analyses, normal distribution was tested for all variables using Shapiro–Wilk tests, showing that 76% of the variables were not normally distributed. Hence, non-parametric tests were used for all uni- and bivariate analyses. Differences between the intervention and control groups were tested using Fisher’s exact test for categorical variables or Mann–Whitney *U* tests for quantitative data. Within-group differences over time were assessed using Wilcoxon matched-pairs signed rank tests. To assess changes over time we calculated the shift for each parameter (baseline [BL] values subtracted from the respective values at time point V1 and V2, shown as ∆V1-BL and ∆V2-BL). Correlations were determined using Spearman's rank coefficient. Multivariate analyses were performed using multiple regressions. Before performing multiple regressions, the variables were tested for intercorrelation (intercorrelations were defined as Spearman’s correlation coefficient > 0.8) showing no significant results. A *p* ≤ 0.07 was considered as a trend, a *p* < 0.05 was considered as statistically significant.

As these analyses are not confirmatory and rather to generate hypotheses to be further analysed in studies with larger populations, e.g. LIBRE-2, we did not perform post-hoc adjustment for multiple testing. Also, in order to try to compensate for random findings in the analyses, we only draw conclusions based on results, which were found (i) consistently for the intervention group and the control group and/or (ii) consistently for both shifts ∆V1-BL and ∆V2-BL. All statistical analyses were performed using GraphPad Prism version 9.1.0 (GraphPad Software, San Diego, CA, USA). Data are shown as medians with interquartile ranges (25th; 75th percentiles).

## Results

### Baseline characteristics

At baseline, the intervention and control groups had similar numbers of women with previously diagnosed breast cancer, vegetarians, and smokers, and did not differ in age, BMI (Table 1), and physical fitness (data not shown).

**Table 1:** Patient characteristics at baseline

Parameters	Intervention group	Control group	*p* value^a^
	(*n* = 33)	(*n* = 35)	
Diseased^b^ [*n* (%)]	23 (69.7)	23 (65.7)	0.799
Vegetarians [*n* (%)]	2 (6.1)	4 (11.4)	0.674
Smokers [*n* (%)]	4 (12.1)	4 (11.4)	0.999
Age [years]	42 (35;49)	41 (35;50)	0.843
BMI [kg/m^2^]	23 (21;28)	24 (21;28)	0.485

*BMI* body mass index. Statistics: Fisher’s exact test (categorical variables) and Mann–Whitney *U* test (numerical data)

^a^Between group difference

^b^Previously diagnosed with breast cancer. Total numbers and percentage (diseased, vegetarians, smokers) or median and interquartile ranges (age, BMI) are shown

At baseline, both groups showed similar adherence to the Mediterranean diet according to the MedD-Score, yet the MEDAS-Score was slightly higher in the intervention group compared to the control group (50% [36%;59%] vs 42% [29%;50%], *p* = 0.045) (Supplementary Table 1). Besides the small difference in the MEDAS-Score, there was no baseline difference in diet. The baseline proportion of eicosanoid acid (20:0) in the plasma fatty acids was higher in the control group compared to the intervention group (0.2% [0.1%;0.2%] vs 0.1% [0.1%;0.2%], *p* = 0.043), while the proportion of linoleic acid (18:2, *n*-6) was higher in the intervention group compared to the control group (30% [26%;32%] vs 28% [26%;30%], *p* = 0.039). All other fatty acids as well as the levels of the intestinal barrier biomarkers LBP and zonulin did not differ between the groups at baseline (Supplementary Table 2).

### The effect of the LIBRE intervention program on dietary and physical outcomes

As described elsewhere [30], adherence to the Mediterranean diet, assessed by both the MEDAS-Score and the MedD-Score, increased markedly in the intervention group for at least 1 year (all *p* < 0.01) (Supplementary Table 1). In the control group, there was a mild but significant increase in the MEDAS-Score and the MedD-Score between baseline and V1 (all *p* < 0.001), which was absent at month 12 (all *p* > 0.05).

**Fig. 1:**
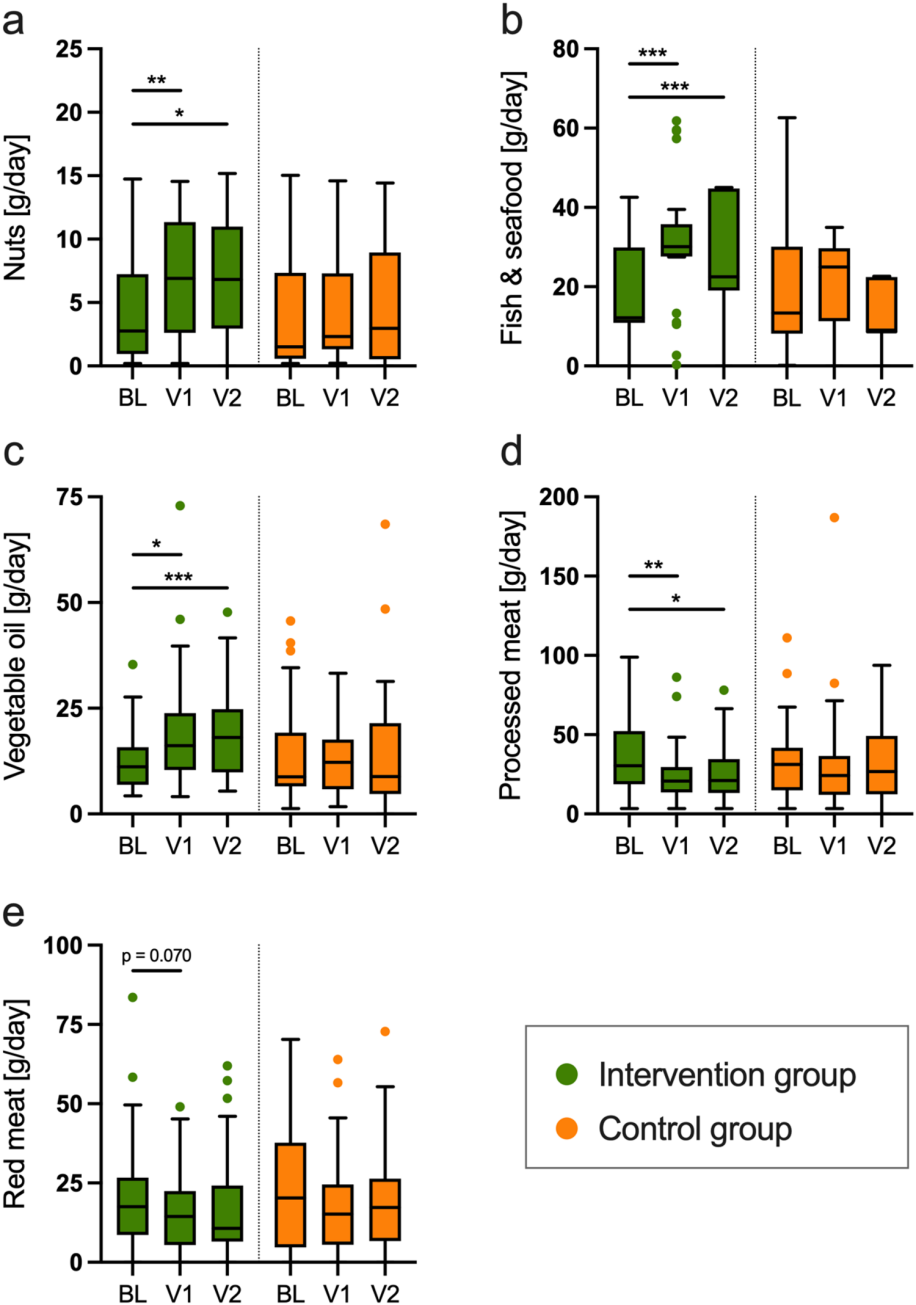
Effect of the intervention on dietary habits. **a**–**e** Shown are data for baseline (BL), as well as after month 3 (V1) and month 12 (V2) for the intake of typical Mediterranean foods. Tukey boxplots with median, whiskers (1.5 × interquartile ranges), and outliers are shown in green (intervention group; *BL: n* = *31, V1, V2: n* = *26*) and orange (control group; *BL: n* = *33, V1: n* = *31, V2: n* = *29*). Within group difference to baseline is indicated by asterisks (*p < 0.05; **p < 0.1; ***p < 0.001; Wilcoxon signed-rank test). This figure summarizes data shown in Supplementary Table 1

Data derived from the EPIC-FFQ showed that participants from the intervention group increased the intake of the typically Mediterranean food groups nuts and seafood (Fig. 1a, b), vegetables, legumes, fruits, olives, and vegetable oil (Fig. 1c) (all *p* < 0.05) (Supplementary Table 1). At the same time, the intake of processed meat (*p* < 0.05 for ∆V1-BL and ∆V2-BL) and red meat (*p* = 0.070 for ∆V1-BL) decreased in the intervention group, but not in the control group (Fig. 1d,e). The participants from the control group decreased the intake of lignin and total diet-derived fat in the first three months and decreased the intake of animal- and plant-derived protein, total fibers, and total diet-derived fat over the 12 month-period (all *p* < 0.05). Dietary data for all time points is shown in Supplementary Table 1.

Physical fitness, assessed objectively in spiroergometry as the ventilatory threshold 1 (VT1), did not change in the intervention group, and showed a slight but significant decrease in the control group between baseline and V1 (−12 W [−62 W;3 W], *p* = 0.020) but not between baseline and V2 (*p* > 0.05). The mean BMI did not change in the intervention group and increased mildly in the control group between baseline and V1 (+ 0.32 kg/m^2^ [− 0.1 kg/m^2^;0.8 kg/m^2^], *p* = 0.013) and increased by trend between baseline and V2 (+ 0.27 kg/m^2^ [− 0.5 kg/m^2^; 0.7 kg/m^2^] *p* = 0.062) (Supplementary Table 1).

### Effect of the Mediterranean diet on plasma fatty acid patterns and intestinal barrier biomarkers

**Fig. 2:**
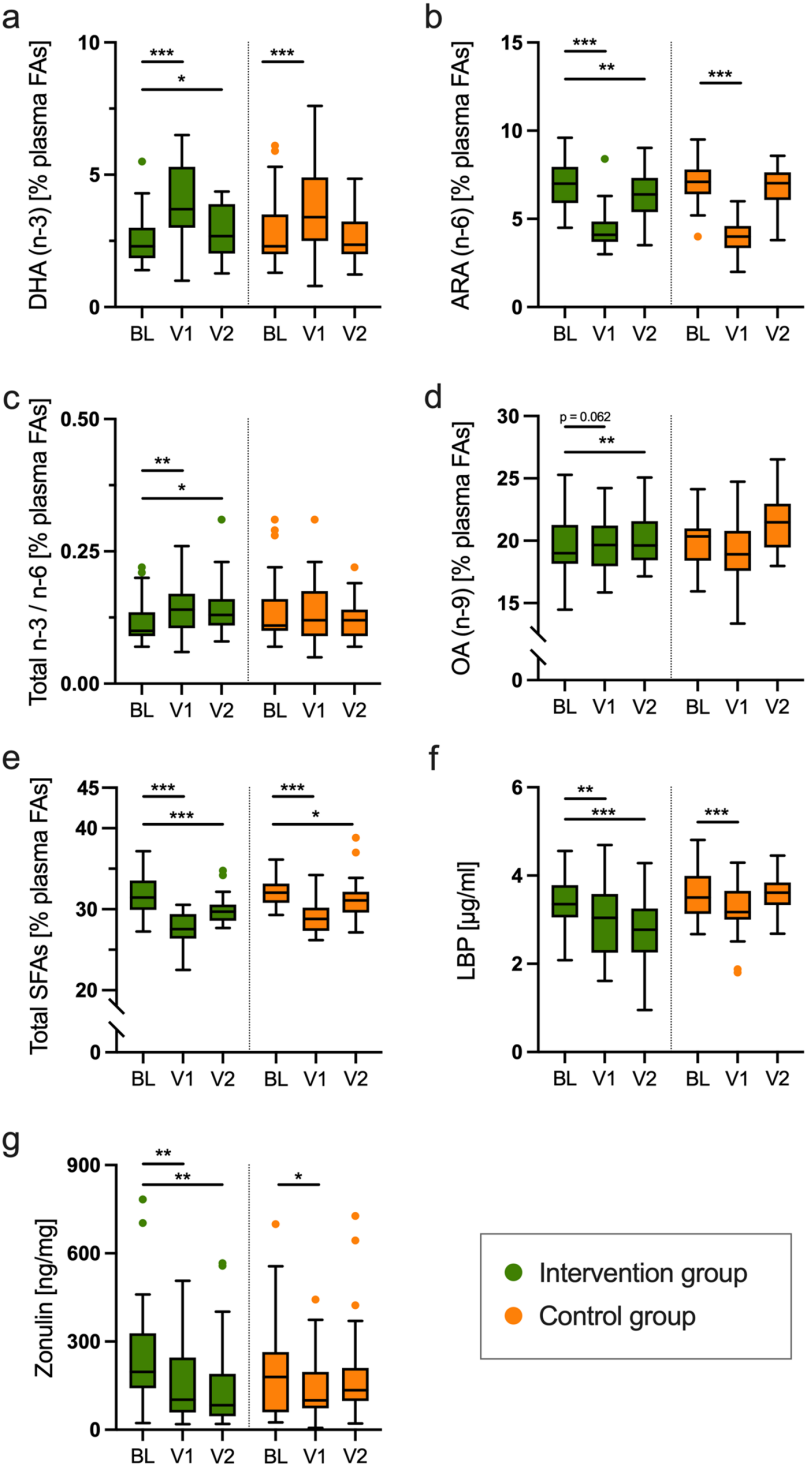
Effect of the intervention on plasma fatty acid composition and intestinal barrier biomarkers Shown are data for baseline (BL), as well as after month 3 (V1) and month 12 (V2) for the proportion (%) of docosahexaenoic acid (DHA), arachidonic acid (ARA), oleic acid (OA), and total saturated fatty acids (SFAs) in the total plasma fatty acid composition (plasma FAs) **(a**–**e),** and for the intestinal barrier biomarkers plasma lipopolysaccharide binding protein (LBP) and fecal zonulin **(f, g)**. Tukey boxplots with median, whiskers (1.5 × interquartile ranges), and outliers are shown in green (intervention group; BL: *n* = 33, V1: *n* = 26–33, V2: *n* = 23–*29*) and orange (control group; *BL: n* = *35, V1: n* = *29–35, V2: n* = *28–32*). Within group difference to baseline is indicated by asterisks (*p < 0.05; **p < 0.1; ***p < 0.001; Wilcoxon signed-rank test). This figure summarizes data shown in Supplementary Table 2

After the 3-month intensive intervention phase, we observed several changes in the plasma fatty acid composition in both study arms. For example, in the first 3 months the proportion of the *n*-3 PUFA DHA (22:6) increased in both groups, while the proportion of the n-6 PUFA arachidonic acid (20:4) decreased (all *p* < 0.001) (Fig. 2a, b). After 1 year, these changes were still present in the intervention group (all *p* < 0.05), but not in the control group. Accordingly, the total *n*-3/*n*-6 ratio increased in the intervention group (all *p* < 0.05) (Fig. 2c). Compared to baseline, the proportion of oleic acid (18:1, *n*-9) increased mildly in the first three months (+ 0.2% [−1.3%;1.2%], *p* = 0.062) and increased markedly after 1 year (+ 1.3% [0.2%;0.4%], *p* = 0.001) in the intervention group (Fig. 2d). There was no change in the proportion of plasma oleic acid in the control group. In both study arms there was a decrease in the proportion of total saturated fatty acids (SFAs) for at least 1 year (intervention group, all *p* < 0.001; control group, all *p* < 0.05) (Fig. 2e).

Both biomarkers of intestinal barrier function decreased in the intervention group in the first 3 months (LBP −0.3 µg/ml [−0.6 µg/ml; 0.1 µg/ml], *p* = 0.007; zonulin −76 ng/mg [−164 ng/mg; −12 ng/mg], *p* = 0.006). In the control group, both biomarkers decreased in the first three months (LBP −0.2 µg/ml [−0.8 µg/ml; −0.1 µg/ml], *p* < 0.001; zonulin −59 ng/mg [−186 ng/mg; 15 ng/mg], *p* = 0.023) but returned to baseline levels after 1 year (Fig. 2f,g). All data on plasma fatty acid composition and the gut barrier biomarkers is shown in detail in Supplementary Tables 1 and 2.

### Omega-3 polyunsaturated fatty acids and intestinal barrier integrity

As a first step, we analyzed correlations between diet, plasma fatty acids, and the intestinal barrier biomarkers. Here, we first calculated the shift for each parameter that was significantly altered during the study (see Supplementary Tables 1 and 2).

As shown in Table 2, the increase in adhering to the Mediterranean diet correlated with the increase in the proportion of DHA and the Omega-3-Index. The increase in the adherence to the Mediterranean diet was furthermore inversely correlated with the proportion of *n*-6 PUFAs. These associations were found consistently in the intervention and control groups. In the intervention group, the MedD-Score correlated with the proportion of the *n*-9 oleic acid at ∆V1-BL.

**Table 2:** Correlation analyses between the shifts in diet, plasma fatty acid composition, intestinal barrier biomarkers, and fecal short-chain fatty acids (SCFAs)

1. Variable	2. Variable	Intervention group		Control group
		∆ V1-BL	∆ V2-BL	∆ V1-BL
∆	∆	*r* (*R*^2^)	*r* (*R*^2^)	*r* (*R*^2^)
MEDAS	*n*-3-Index		0.547 (0.30)*	0.417 (0.17)*
MedD	*n*-3/*n*-6 ratio	0.512 (0.26)**	0.535 (0.29)*	
MedD	22:6	0.404 (0.16)*	0.710 (0.50)**	0.383 (0.15)*
MedD	Total *n*-6	−0.460 (0.21)*		−0.402 (0.16)*
MedD	18:1	0.431 (0.19)*		
MEDAS	Total SFAs	−0.477 (0.23)*		
Nuts	22:6	0.415 (0.17)*	0.487 (0.24)*	
Fish	22:6	0.408 (0.17)*		
Nuts	20:2	−0.484 (0.23)*		
Veg. oils	18:1		0.670 (0.45)**	
Proc. meat	Total SFAs	0.422 (0.18)*	0.481 (0.23)*	
LBP	DHA	**−0.368 (0.14)**^**#**^		−0.570 (0.32)**
LBP	*n*-3-Index			−0.507 (0.26)**
Zonulin	DHA	−0.426 (0.18)*	−0.463 (0.21)*	
Zonulin	*n*-3-Index	**−0.394 (0.16)**^**#**^	−0.481 (0.23)*	
Zonulin	*n*-3/*n*-6 ratio	0.450 (0.20)^**#**^		
LBP	Total SFAs	0.574 (0.33)**	0.430 (0.18)*	0.371 (0.14)*
LBP	Butyrate	−0.748 (0.56)***	−0.752 (0.57)***	
LBP	Propionate	−0.731 (0.53)***	−0.739 (0.55)***	
Zonulin	Butyrate	−0.632 (0.40)***		−0.719 (0.52)***
Zonulin	Propionate	−0.611 (0.37)***		−0.736 (0.54)***

Shown are the data for the shift between baseline (BL) and month 3 (∆ V1-BL) and for the shift between BL and month 12 (∆ V2-BL). Only correlations with parameters which changed during the study are shown in this table (see Supplementary Tables 1 and 2)

Statistics: Spearman correlation (^#^*p* ≤ 0.070; *p < 0.050; **p < 0.010; ***p < 0.001). Intervention group (∆ V1-BL: *n* = 25–26; ∆ V2-BL: *n* = 21–26), control group (∆ V1-BL: *n* = 29–31; ∆ V2-BL: *n* = 27–29). Further abbreviations: *DHA* docosahexaenoic acid; *LBP* lipopolysaccharide binding protein; *MEDAS* Mediterranean Diet Adherence Screener; *MedD* Mediterranean Diet Score; *n* Omega; *n-3-Index* Omega-3-Index; *SCFAs* short-chain fatty acids; *SFA* saturated fatty acids; *Veg* vegetables. Correlations which were found (i) consistently for the intervention group and the control group and/or (ii) consistently for the two shifts ∆V1-BL and ∆V2-BL are highlighted in boldface. There were no significant results in the control group for ∆ V2-BL

**Fig. 3:**
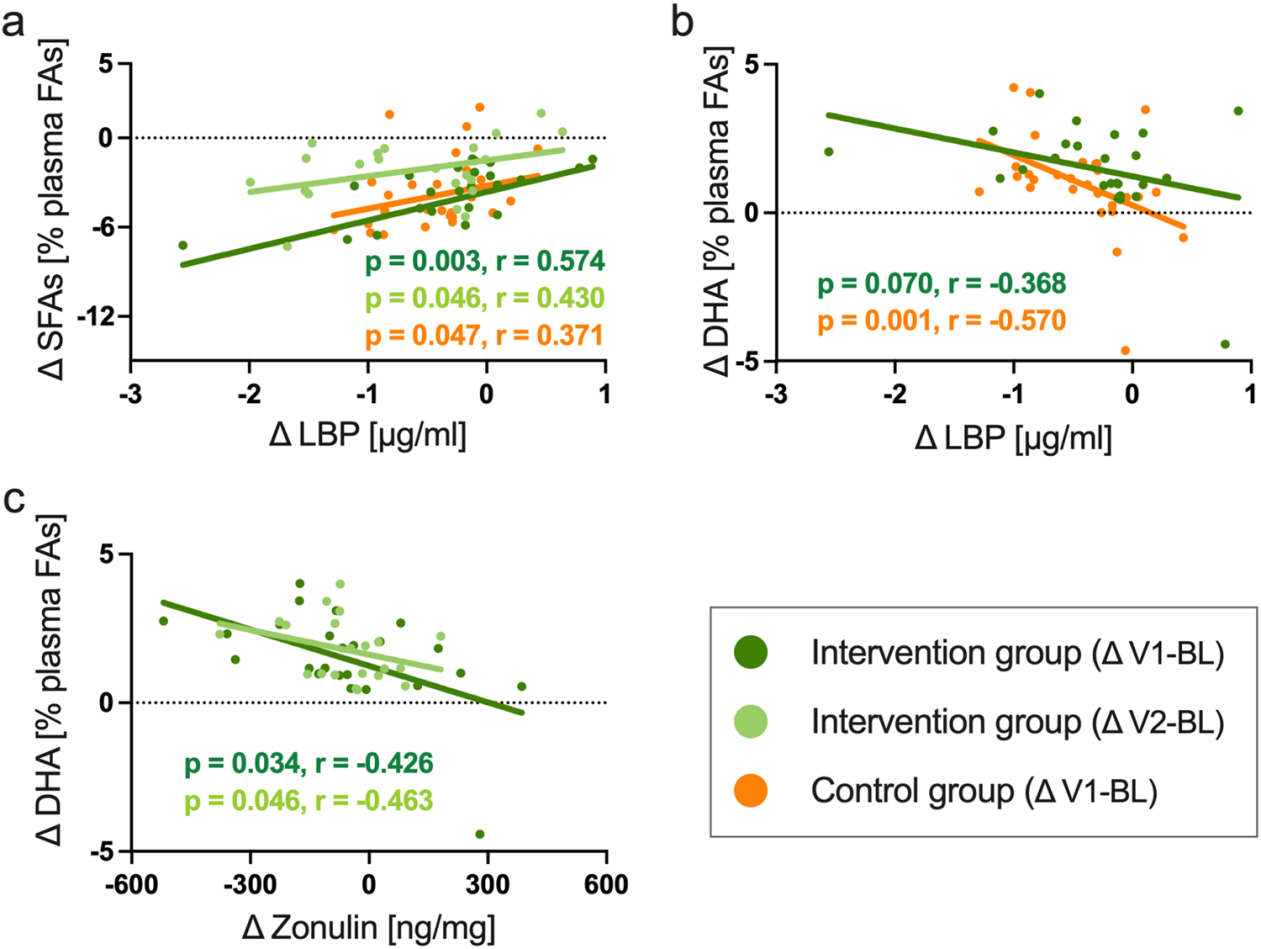
Omega-3 polyunsaturated fatty acids improve intestinal barrier integrity. Shown are the correlations between the proportion (%) of saturated fatty acids (SFAs) in the total plasma fatty acid composition (plasma FAs) and plasma levels of lipopolysaccharide binding protein (LBP) (**a**); the proportion of the omega-3 polyunsaturated docosahexaenoic acid (DHA) and plasma levels of LBP (**b**); and the proportion of DHA and fecal levels of zonulin (***c***). Spearman correlations were conducted for the intervention and the control groups (*n* = 33/35) using shift values (baseline [BL] values subtracted from the respective values after month 3 [V1] and month 12 [V2]. This figure summarizes the main findings shown in detail in Table 2

The intake of nuts was associated with the proportion of DHA, and the intake of processed meat was associated with the proportion of SFAs. These associations were found consistently for both ∆V1-BL and ∆V2-BL in the intervention group. The intake of vegetable oils correlated strongly with the proportion of oleic acid in the intervention group for ∆V2-BL but not the other time points. As shown in Fig. 3a, the decrease in the proportion of SFAs correlated with the decrease in the plasma levels of the intestinal barrier biomarker LBP for both ∆V1-BL and ∆V2-BL in the intervention group as well as for ∆V1-BL in the control group. Also, the increase in the proportion of DHA correlated with the decrease in plasma LBP (Fig. 3b) and fecal zonulin (Fig. 3c). All results from the correlation analyses are shown in Table 2.

To further investigate the observed associations between the plasma fatty acid composition and the intestinal permeability biomarkers, we ran multiple regression models, including BMI, physical fitness, and previous cancer disease state as possible confounders. As shown in Supplementary Table 3, these multivariate analyses confirmed the initial correlation analyses to a large degree. In detail, the association between the proportion of SFAs and plasma LBP was also significant in the intervention group for ∆ V1-SE (*p* = 0.048, *R*^2^ = 0.45), but was not significant at the other time points and not in the control group. Furthermore, the multivariate analyses confirmed the inverse association between the proportion of DHA and plasma LBP for ∆ V1-SE both in the intervention and the control groups (intervention group: *p* = 0.070, *R*^2^ = 0.42; control group: *p* = 0.023, *R*^2^ = 0.37). Also, the regression models confirmed the inverse association between the proportion of DHA and fecal zonulin in the intervention group (**∆** V1-BL: *p* = 0.049, *R*^2^ = 0.40; **∆** V2-BL: *p* = 0.040, *R*^2^ = 0.42).

Taken together, the initial correlation analyses as well as the subsequent multivariate analyses showed an inverse association between the proportion of DHA in the plasma fatty acids and the two intestinal barrier biomarkers LBP and zonulin. Furthermore, the results showed an association between the proportion of SFAs and LBP.

### Intestinal short-chain fatty acids have a greater effect on intestinal barrier integrity than plasma omega-3 polyunsaturated fatty acids

We have previously shown that fecal short-chain fatty acids (SCFAs) are key mediators for the favorable effects of the Mediterranean diet on intestinal barrier integrity [5]. In this previous article we showed strong inverse associations between fecal levels of the SCFAs propionate and butyrate and the intestinal barrier biomarkers plasma LBP and fecal zonulin.

As a final step in the present analysis, we compared the effect size of the association between the proportion of DHA in the plasma fatty acids and the two intestinal barrier biomarkers (plasma LBP and fecal zonulin) with the effect size of the association between fecal SCFAs (propionate and butyrate) and the two intestinal barrier biomarkers.

**Fig. 4:**
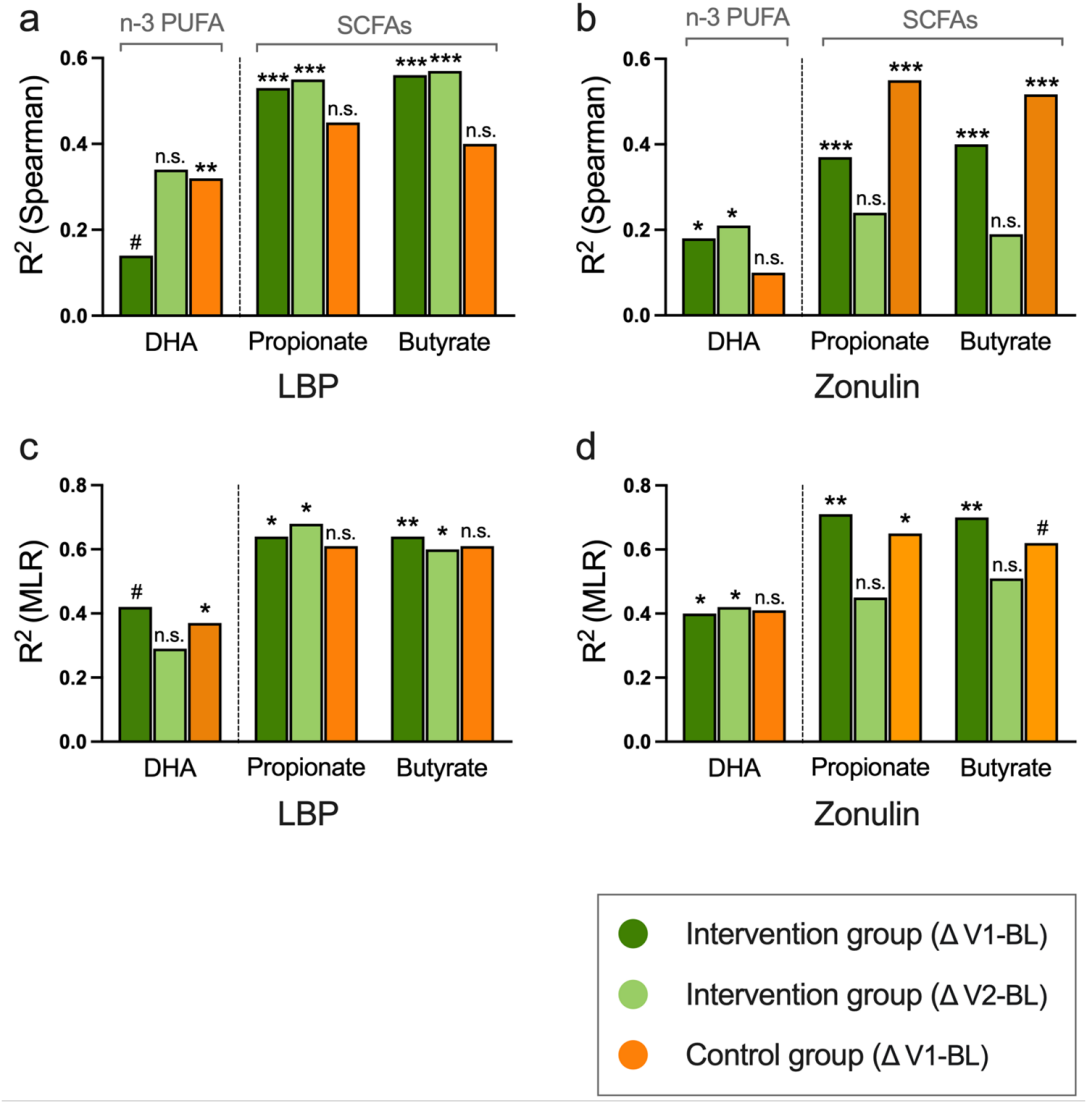
Omega-3 polyunsaturated fatty acids improve intestinal barrier integrity—albeit to a lesser degree than fecal short-chain fatty acids. Shown are the comparisons of the effect sizes (*R*^2^) of the correlation between the proportion (%) of the omega-3 polyunsaturated fatty acid (*n*-3 PUFA) docosahexaenoic acid (DHA) in the total plasma fatty acid composition and the fecal short-chain fatty acids (SCFAs) propionate and butyrate with plasma lipopolysaccharide binding protein (LBP) (**a**), and fecal zonulin (**b**). Panels **c**, **d** show the comparisons of the effect sizes of the multiple linear regressions (MLR) between the proportion of DHA and the fecal SCFAs propionate and butyrate with plasma LBP (**c**) and fecal zonulin (**d**). #*p* < 0.07; *p < 0.05; **p < 0.01; ***p < 0.001; *n.s.* not significant. This figure summarizes the main findings shown in detail in Table 2 and Supplementary Table 3

First, we ran correlation analyses, including the fecal amounts of the SCFAs propionate and butyrate (mg SCFA/g fecal sample) and the levels of the intestinal barrier biomarkers plasma LBP and fecal zonulin. As shown in Table 2 and summarized in Fig. 4a, b, the effect size (*R*^2^) was markedly higher for the association between fecal SCFAs and the intestinal barrier biomarkers (*R*^2^ 0.37–0.57; all *p* < 0.0001) compared to the effect size of the association between the proportion of DHA in the plasma fatty acids and the intestinal barrier biomarkers (*R*^2^ 0.14–0.32; all *p* ≤ 0.070).

Subsequently, we ran multivariate regression analyses to verify our findings from the correlation analyses with BMI, physical fitness, and previous cancer disease state as possible confounders. As summarized in Fig. 4c, d, these multivariate analyses supported the results from the correlation analyses, showing that fecal SCFAs had a more pronounced effect on the intestinal barrier biomarkers than the proportion of DHA (SCFAs and barrier biomarkers: *R*^2^ 0.60–0.71; DHA and barrier biomarkers: *R*^2^ 0.37–0.42; all *p* ≤ 0.070). All results from the multiple regression models are shown Supplementary Table 3.

## Discussion

Intestinal barrier dysfunction is a major cause that drives low-grade inflammation found in numerous chronic diseases, including cardiometabolic diseases like type 2 diabetes, and cancer [2, 36]. Adherence to the Mediterranean diet, on the other hand, shows beneficial effects on these chronic diseases [14–18]. In the present study, we show that an increase in the proportion of n-3 PUFAs in the plasma fatty acids, derived from adherence to the Mediterranean diet, improves intestinal barrier dysfunction.

In a randomized controlled trial, we assessed the effect of a 1-year Mediterranean diet on plasma fatty acid levels and intestinal barrier integrity in a cohort of women with mild barrier impairment due to BRCA germline mutations [5, 20]. Our data show that adherence to the Mediterranean diet changes the plasma fatty acid composition, increasing the proportion of anti-inflammatory *n*-3 PUFAs and *n*-9 MUFAs, while decreasing the proportion of SFAs and pro-inflammatory *n*-6 PUFAs.

Thus far, it has been largely unclear how adherence to the Mediterranean diet exerts its beneficial effects on chronic diseases. Preclinical studies suggested that diet-derived organic acids like ferulic acid found in grains [37], but especially *n*-3 PUFAs might be of major relevance here. For the first time in a clinical setting, we show that *n*-3 PUFAs improve intestinal barrier function, which has so far only been shown in cell lines and animal models. Using bi- and multivariate analyses we found that *n*-3 PUFAs, especially DHA found in fat fish like salmon, improved intestinal barrier function, while SFAs, found in confectionery and fast food, were associated with barrier dysfunction. Of note, we found no association between the proportion of *n*-6 PUFAs or *n*-9 MUFAs in the plasma fatty acids and the two barrier biomarkers, indicating that an effect of omega fatty acids on barrier integrity is exclusive for *n*-3 PUFAs.

Marine-derived *n*-3 PUFAs like eicosapentaenoic acid (EPA) and DHA are incorporated into cell membranes, including intestinal epithelial cell membranes, and exert several biological effects. The best-known mechanisms comprise the induction of anti-inflammatory eicosanoids derived from EPA and docosanoids derived from DHA [38], which are linked to lower cancer incidence [39]. Also, recent studies showed that marine-derived *n*-3 PUFAs improve intestinal barrier function, which has been shown in cell lines [40–47] and rodents [43, 48–56].

According to in vitro and in vivo studies, *n*-3 PUFAs affect tight junction proteins, including occludin and zonula occludens-1 (ZO-1), which are essential for effective cell–cell connections, which are necessary to prevent uncontrolled paracellular permeability. In detail, in vitro studies [44–46] and rodent models [50, 51, 57] showed that long-chain *n*-3 PUFAs (eicosapentaenoic acid and DHA) improved gut barrier stability via an increased expression of occludin and ZO-1 in cell membranes and decreased cellular degeneration. Furthermore, *n*-3 PUFAs induce the G-protein coupled receptor 120, which exerts anti-inflammatory effects and increases tight junction stability [10, 58, 59]. For the first time in a clinical setting, our data show a barrier-stabilizing effect of *n*-3 PUFAs in humans, using two validated barrier biomarkers LBP and zonulin.

Intestinal barrier function is affected by numerous exogenous and endogenous factors, including lifestyle factors like diet, physical activity, alcohol intake or smoking, but also gut microbiota composition and function [2, 3, 60]. As shown by our data, *n*-3 PUFAs have a significant influence on barrier integrity. However, barrier-stabilizing effects are not limited to *n*-3 PUFAs. Early and recent research showed that SCFAs, derived from bacterial fermentation of dietary fibers in the colon, improve intestinal barrier function [2, 60, 61], exert anti-inflammatory effects [62, 63], and might regulate cancer progression [64]. In the present study, we assessed fecal SCFA levels to compare the effect size of *n*-3 PUFAs on intestinal barrier integrity with the effect size of SCFAs on barrier integrity. To the best of our knowledge, this is the first clinical study to investigate this. Our data suggest that the effect of fecal SCFAs on barrier function is more pronounced than the effect of *n*-3 PUFAs on barrier function. Obviously, the LIBRE study design does not allow to draw conclusions regarding the underlying molecular mechanisms, as extensive phenotyping would be necessary to do so. We assume, however, that several aspects play a role here. Most importantly, SCFAs are produced in the colon and are metabolized to a large degree by enterocytes where SCFAs directly improve barrier integrity [2, 60]. On the other hand, *n*-3 PUFA intake is regulated in proximal parts of the small intestine. Therefore, only smaller amounts of *n*-3 PUFAs reach enterocytes via the bloodstream which might lessen the effect size of *n*-3 PUFAs. Future studies should assess whether *n*-3 PUFAs applicated rectally or via capsules which release *n*-3 PUFAs in more distal parts of the intestinal tract increase the effect of these PUFAs on intestinal barrier function. Furthermore, the effect of food components other than fatty acids, fibers, and SCFAs, e.g. vitamins, minerals, amino acids and polyphenols, which might also affect intestinal barrier integrity, should be evaluated [8, 9].

### Limitations and strengths

Our study has both limitations and strengths. A limitation of our study is that we only included women with BRCA mutations, which implies an associated mild intestinal barrier dysfunction. To what extent our finding will also hold true for other populations with or without intestinal barrier dysfunction needs to be explored in future studies. A strength of our study is that our findings on the effects of omega-3 polyunsaturated fatty acids in the Mediterranean diet on intestinal barrier function is based on a rigorous approach in the statistical analyses. To omit reporting random findings, we only show results which were found consistently in the two study groups and/or found for more than one time point.

In conclusion, our data show that *n*-3 PUFAs, derived from typical Mediterranean foods like fatty fish, improve intestinal barrier function. However, the effect of gut bacteria-derived SCFAs on intestinal barrier function was more pronounced than the effect of diet-derived *n*-3 PUFAs. Our study offers new insights in the interplay between dietary components and intestinal health.

## Supplementary Information

Below is the link to the electronic supplementary material.

Supplementary file1 (PDF 187 KB)

## Data Availability

Data described in the manuscript will be made available upon request pending approval by the corresponding author SCB

## Declarations

### Clinical trial registration

The trial has been registered at ClinicalTrials.gov (reference number NCT02087592).
