# Supplementary material for: Omega-3 polyunsaturated fatty acids improve intestinal barrier integrity—albeit to a lesser degree than short-chain fatty acids: an exploratory analysis of the randomized controlled LIBRE trial
Source: Eur J Nutr. 2023 Jun 15;62(7):2779–91. doi: 10.1007/s00394-023-03172-2 (PMC10468946; doi:10.1007/s00394-023-03172-2)
Supplement: Supplementary file 1 — Supplementary file1 (PDF 187 KB) [file 394_2023_3172_MOESM1_ESM.pdf]

Omega-3 polyunsaturated fatty acids improve intestinal barrier integrity - albeit to a lesser degree than short-chain fatty acids: an exploratory analysis of the randomized controlled LIBRE trial

Benjamin Seethaler[1], Katja Lehnert[2], Maryam Yahiaoui-Doktor[3], Maryam Basrai[1], Walter Vetter[2], Marion Kiechle[4], Stephan C. Bischoff[1]

European Journal of Nutrition

[1]Institute of Nutritional Medicine, University of Hohenheim, Stuttgart, Germany

[2]Institute of Food Chemistry, University of Hohenheim, Stuttgart, Germany

[3]Institute for Medical Informatics, Statistics and Epidemiology (IMISE), University of Leipzig, Leipzig, Germany

[4]Department of Gynecology, Center for Hereditary Breast and Ovarian Cancer, Klinikum Rechts der Isar, Technical University Munich and Comprehensive Cancer Center Munich, Munich, Germany

Corresponding author: Stephan C. Bischoff, M.D., Professor of Medicine, Institute of Nutritional Medicine, University of Hohenheim, Fruwirthstr. 12, 70593 Stuttgart, Germany. Phone: +4971145924101. E-mail: bischoff.stephan@uni-hohenheim.de

<u>Supplementary material</u>

Supplementary Figure 1 Page 2

Supplementary Table 1 Pages 3,4

Supplementary Table 2 Pages 5,6

Supplementary Table 3 Page 7

Supplementary Figure 1

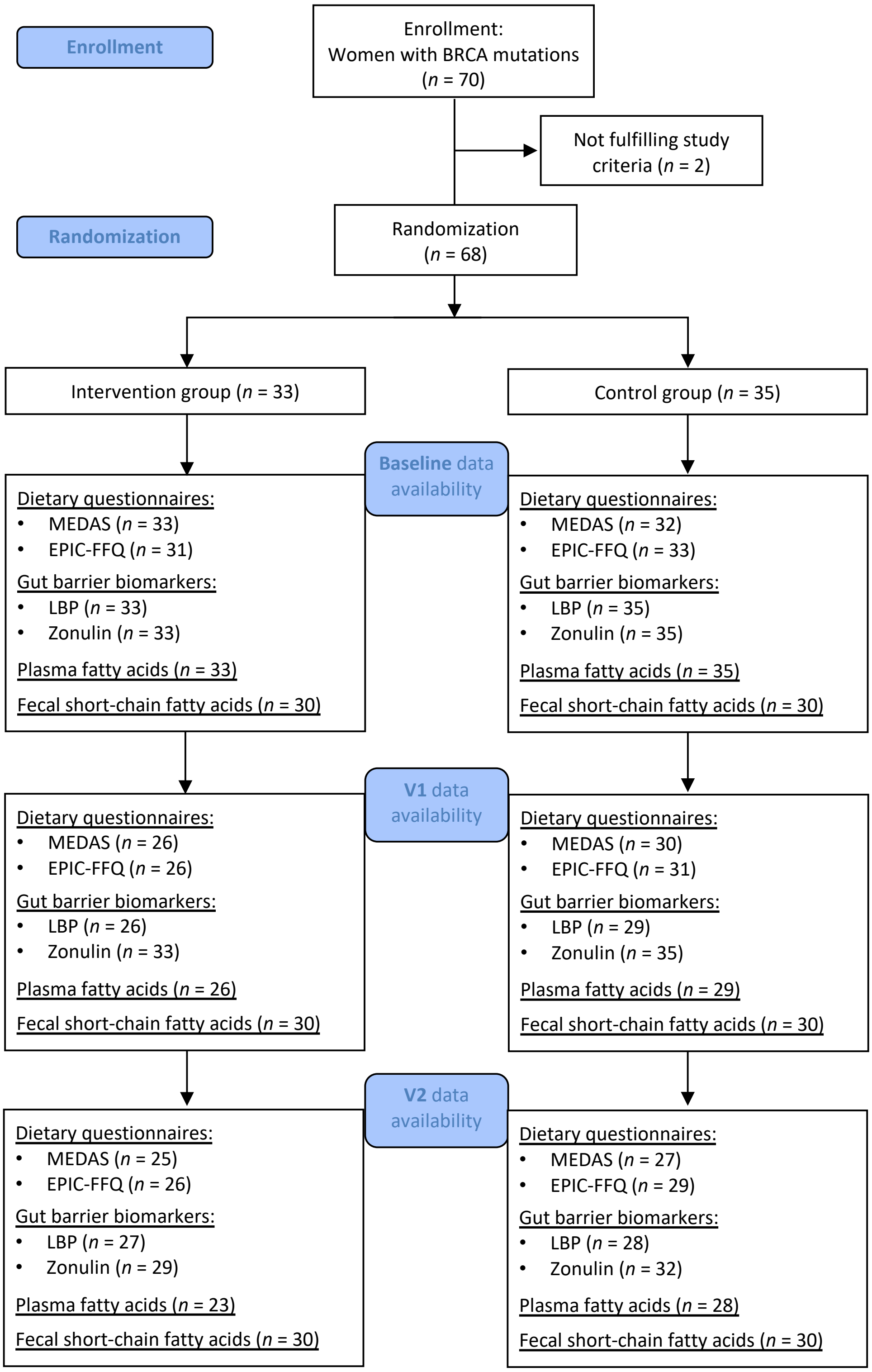

CONSORT flow diagram showing data availability for each study visit. Abbreviations: BRCA, breast cancer gene; MEDAS, Mediterranean Diet Adherence Screener; EPIC-FFQ, European Prospective Investigation into Cancer and Nutrition – Food Frequency Questionnaire; LBP, lipopolysaccharide binding protein; V1, 3 months after baseline; V2, 12 months after baseline.

Supplementary Table 1. Changes in dietary behaviour during the study. Shown are the adherence to the Mediterranean diet (MedD), the intake of nutrients and food groups according to the Food Frequency Questionnaire (FFQ), and the body mass index (BMI) for baseline (BL), for the shift between BL and month 3 (Δ V1-BL), and for the shift between BL and month 12 (Δ V2-BL).

	Intervention group			Control group			Intervention vs. control groups (p)	
	BL (n = 31-33)	Δ V1-BL (n = 26)	Δ V2-BL (n = 22-26)	BL (n = 32-35)	Δ V1-BL (n = 30-31)	Δ V2-BL (n = 27-29)	Δ V1-BL	Δ V2-BL
**MedD-scores**								
MEDAS [%]	50 (36;59)	**7.1 (0;29)****	**14 (0;21)****	42 (29;50)[#]	**7.1 (0;21.43)*****	0 (0;14)	0.549	0.215
MedD [points]	4 (4;5)	**1.5 (0.8;3)*****	**1 (-0.3;2.3)****	4 (3;6)	**1 (0;2)*****	0 (-1;2)	0.527	0.083
**Nutrients [g/d]**								
Plant protein	25 (22;31)	1.1 (-3.9;6.5)	2.7 (-0.7;7.5)	26 (21;32)	-0.7 (-3.2;3.1)	**-2.3 (-8.6;1.5)***	0.302	**0.003**
Animal protein	40 (31;50)	-0.9 (-9;6)	0.4 (-9;8)	45 (30;51)	-2.6 (-8;7)	**-5.2 (-13;-0.1)****	0.732	0.090
Cellulose	4.3 (3.7;5.1)	0.3 (-0.3;1.4)	**0.7 (-0.1;1.4)***	4.7 (3.8;5.6)	-0.3 (-1.1;0.6)	-0.4 (-1.2;0.4)	0.060	**0.002**
Lignin	1.3 (1;2)	0 (-0.3;0.2)	0.2 (-0.1;0.4)	1.4 (1.1;1.8)	**-0.1 (-0.4;0.1)***	-0.1 (-0.5;0.2)	0.163	0.052
Waterinsol. fibers	14 (11;18)	0.8 (-1.5;3.6)	**2.5 (-0.7;4.3)***	15 (12;18)	0.0 (-2.6;1.3)	**-1.4 (-3.6;0.8)***	0.050	**0.002**
Watersol. fibers	6.9 (5.6;8.6)	0.5 (-1;2.1)	**1.2 (0;2.7)***	7.0 (5.5;8.4)	0.0 (-1.5;0.8)	**-0.7 (-2.2;0.3)***	0.138	**0.001**
Polysaccharides	97 (80;130)	1.5 (-25;22)	5.0 (-8.4;39)	98 (87;137)	-1.0 (-23;8)	-13.3 (-29.3;12)	0.530	**0.021**
Non res. oligos.	0.3 (0.2;0.4)	**0.1 (0;0.2)***	0.1 (0;0.1)	0.3 (0.2;0.4)	0.0 (-0.1;0.1)	0.0 (-0.1;0.0)	**0.012**	**0.021**
Res. oligos.	1.2 (0.9;2.4)	0.0 (-0.1;0.7)	0.1 (-0.2;0.8)	1.1 (0.7;3.1)	0.0 (-0.2;0.3)	0.1 (-0.3;0.6)	0.431	0.506
Fibers	21 (17;26)	1.3 (-2.5;6.1)	**4.0 (-0.4;7.5)***	21 (18;26)	-0.3 (-4.0;2.0)	**-2.1 (-6.3;0.9)***	0.072	**0.001**
Fat	88 (75;102)	5.7 (-8.8;20)	5.2 (-2.4;11)	100 (85;115)	**-8.7 (-23;4)***	**-10.7 (-36;3)***	**0.036**	**0.001**
Carbohydrates	200 (160;248)	4.1 (-22;50)	17 (-18;34)	244 (188;276)	-9.7 (-40;15)	-19 (-77;16)	0.104	**0.020**
**Food groups [g/d]**								
Potatoes	34 (22;47)	-0.1 (-11;17)	0.1 (-4.2;2.9)	45 (34;67)	-0.6 (-19;11)	0.1 (-25.9;11.7)	0.421	1.000
Vegetables	131 (105;186)	**36 (-2.6;74)***	**26 (-14;69)***	133 (89;193)	-8.0 (-29.8;54)	-4.0 (-45;30)	0.101	0.072
Legumes	2.7 (1.7;4.8)	**1.8 (-0.6;8.9)****	**2.6 (-0.3;5.2)****	3.3 (1;4.9)	0.6 (-1.7;3)	0.4 (-2.3;2.5)	0.094	0.087
Fruits	176 (131;256)	**43 (-8;137)***	**55 (-1.3;119)****	211 (128;288)	-11 (-79.6;73)	12 (-59.8;69)	**0.027**	0.116
Nuts	2.8 (1;7.2)	**1.8 (-0.3;4.9)****	**2.4 (-0.3;6.2)***	1.5 (0.6;7.3)	0.2 (-0.7;1.7)	0.1 (-0.8;2.8)	0.051	0.091
Olives	0.2 (0.1;0.4)	0.1 (-0.2;0.3)	**0.2 (0;0.5)*****	0.2 (0;0.6)	0.0 (-0.1;0.3)	0 (-0.1;0.4)	0.814	0.107
Milk	125 (61;232)	11 (-16;71)	-2.1 (-62;50)	115 (52;214)	0.0 (-28;95)	-0.4 (-32;29)	0.784	0.552
Yoghurt	55 (19;88)	3.5 (-24;44)	-3.9 (-47;17)	41 (18;72)	0.0 (-21;18)	0.0 (-13;20)	0.305	0.228
Cereals	177 (142;250)	-5.1 (-56;53)	17 (-17;89)	176 (140;265)	-2.8 (-44;30)	-35 (-75;32)	0.905	**0.014**

Suppl. Table 1 (continued)	**Intervention group**			**Control group**			**Intervention vs. control groups (p)**	
	**BL (n = 31-33)**	**∆ V1-BL (n = 26)**	**∆ V2-BL (n = 22-26)**	**BL (n = 32-35)**	**∆ V1-BL (n = 30-31)**	**∆ V2-BL (n = 27-29)**	**∆ V1-BL**	**∆ V2-BL**
**Food groups [g/d]**								
Red meat	18 (8.7;27)	-2.6 (-14;0.8)	-1.9 (-15;5.1)	20 (4.7;38)	-1.8 (-14;1.4)	-1.2 (-17;2.3)	0.647	0.808
Poultry	8.9 (2.8;20)	-0.6 (-12;3.1)	0.4 (-12;13)	10.6 (3.3;22)	-0.5 (-7.0;3.7)	-4.5 (-11;1)	0.433	0.252
Processed meat	30 (19;52)	**-8.7 (-13;-0.3)****	**-4.3 (-15;3.5)***	31 (15;42)	-1.6 (-12;4.3)	0.9 (-9;6.1)	0.091	0.295
Fish	8.7 (8.6;22)	**11 (0;14)*****	**14 (0;22)*****	8.7 (1.9;22)	0.0 (0.0;7.0)	0.0 (-7.6;4)	0.066	**0.002**
Fish & seafood	12 (11;30)	**14 (-0.3;21)*****	**18 (0;26)*****	13 (8;30)	0.0 (-2.2;11)	0.0 (-9;4)	**0.043**	**0.002**
Egg	10 (7.1;16)	0.5 (-1.8;3.8)	**4.4 (-0.7;8.6*)**	10 (4.8;14)	-0.2 (-3.1;4.6)	0.9 (-2.5;4.8)	0.489	0.197
Vegetable oils	11 (6.9;16)	**4.4 (-1;15)***	**4.9 (-0.1;12)*****	9.0 (6.5;19)	0.5 (-2.8;7.0)	0.5 (-7.7;9)	0.127	**0.034**
Butter	6.6 (3.2;12)	-0.1 (-3.1;1.1)	0.5 (-2;6.1)	9.2 (2.6;14)	-0.1 (-3.5;3.3)	-0.2 (-5.2;3.7)	0.509	0.352
Confectionery	35 (21;59)	-2.1 (-18;4.9)	**-5.4 (-14;0.7)****	50 (28;65)	-5.6 (-18;7.2)	-4 (-22;7.2)	0.843	0.913
Wine	46 (7.5;90)	0.0 (-16;38)	3.5 (-4.2;55)	30 (10.8;108)	0.0 (-13;4.0)	0.0 (-17.3;67)	0.238	0.522
**BMI**								
BMI [kg/m^2]	23 (21;28)	-0.2 (-0.7;0.1)	0.1 (-0.6;0.7)	23 (21;28)	**0.32 (-0.1;0.81)***	0.27 (-0.48;0.72)	**0.002**	0.553

Median and interquartile ranges (25th;75th percentiles) are shown. Difference between the study groups at baseline and difference between the group's shifts was tested using the Mann-Whitney *U* test ($^{\#}$difference between the groups at baseline; p < 0.05). Within group difference between baseline and V1/V2 was tested using the Wilcoxon signed-rank test (*p < 0.05; **p < 0.01; ***p < 0.001). Further abbreviations: MEDAS-Score, Mediterranean Diet Adherence Screener-Score; MedD-Score, Mediterranean Diet Score; oligos., oligosaccharides; res., resorbable; watersol., watersoluble; waterins., waterinsoluble.

Supplementary Table 2. Changes in plasma fatty acid patterns and the intestinal barrier biomarkers plasma lipopolysaccharide binding protein (LBP) and fecal zonulin during the study. Shown are the data for baseline (BL), for the shift between BL and month 3 (Δ V1-BL), and for the shift between BL and month 12 (Δ V2-BL).

	**Intervention group**			**Control group**			**Intervention vs. control groups (p)**	
	**BL (n = 33)**	**Δ V1-BL (n = 26-33)**	**Δ V2-BL (n = 23-29)**	**BL (n = 35)**	**Δ V1-BL (n = 29-35)**	**Δ V2-BL (n = 28-32)**	**Δ V1-BL**	**Δ V2-BL**
**Fatty acids [%]**								
12:0	0.1 (0.1;0.2)	**-0.1 (-0.2;-0.1)*** **	0.0 (-0.1;0.1)	0.1 (0.1;0.2)	**-0.1 (-0.1;-0.1)*** **	0.0 (-0.1;0)	0.167	0.640
13:0	0.1 (0.1;0.1)	**0.1 (0;0.1)*** **	**0.1 (0;0.1)* **	0.1 (0;0)	**0.1 (0;0.1)*** **	0.0 (0;0)	0.119	0.374
14:0	1.2 (0.9;1.6)	-0.1 (-0.3;0.2)	0.0 (-0.4;0.3)	1.2 (1;1.4)	-0.1 (-0.3;0.1)	0.1 (-0.3;0.4)	0.823	0.758
15:0 i/a	0.1 (0;0.1)	0.0 (0.01;0)	0.0 (0;0.01)	0.1 (0;0.01)	0.0 (0;0)	0.0 (0;0)	0.863	0.431
15:0	0.2 (0.2;0.3)	**-0.1 (-0.1;0)*** **	0.0 (0;0)	0.2 (0.2;0.3)	0.0 (-0.1;0)	0.0 (0;0)	0.770	0.758
16:0 i	0.1 (0;0.1)	**0.1 (0;0.1)*** **	0.0 (0;0)	0.1 (0;0.1)	**0.1 (0;0.1)*** **	0.0 (0;0)	0.770	0.454
16:0	21 (19;23)	**-1.6 (-3.6;-0.6)*** **	-0.6 (-2.2;1.1)	21 (20;23)	**-1.4 (-2.2;0.2)*** **	0.1 (-0.8;1.3)	0.240	0.164
17:0 i	0.2 (0.1;0.2)	0.0 (0;0)	0.0 (0;0)	0.1 (0.1;0.2)	0.0 (0;0)	0.0 (0;0.1)	0.352	0.454
17:0 a	0.1 (0.1;0.1)	0.0 (0;0)	0.0 (0;0)	0.1 (0.1;0.1)	0.0 (0;0)	0.0 (0;0.1)	0.705	0.913
17:0	0.3 (0.2;0.3)	-0.1 (-0.1;0)	0.0 (0;0)	0.3 (0.3;0.3)	**-0.1 (-0.1;0)*** **	0.0 (-0.1;0)	0.744	0.727
18:0	7.3 (6.8;7.7)	**-0.7 (-1.2;-0.2)** **	**-0.3 (-0.7;0)** **	7.4 (6.8;7.6)	**-0.5 (-1.2;-0.2)*** **	-0.1 (-0.8;1)	0.823	0.276
20:0[1]	0.1 (0.1;0.2)	**-0.1 (-0.1;0)*** **	0.0 (-0.1;0)	0.2 (0.1;0.2)[#]	**-0.1 (-0.1;-0.1)*** **	**-0.1 (-0.1;0)* **	0.177	0.364
20:0[2]	0.1 (0.1;0.1)	**-0.1 (-0.1;0)*** **	0.0 (0;0)	0.1 (0.1;0.1)	**-0.1 (-0.1;0)** **	0.0 (0;0)	0.850	1.000
22:0	1.3 (0.9;1.4)	**-1.2 (-1.3;-0.8)*** **	**-1.3 (-1.4;-0.9)*** **	1.4 (1.1;1.5)	**-1.3 (-1.5;-1.1)*** **	**-1.3 (-1.5;-1.1)*** **	0.107	0.442
14:1 n-5	0.1 (0;0.1)	0.0 (0;0)	0.0 (0;0)	0.1 (0;0.1)	0.0 (0;0)	0.0 (0;0)	0.904	0.866
16:1 n-7	2.4 (1.6;2.9)	-0.1 (-0.7;0.3)	0.3 (-0.2;0.6)	2.5 (1.7;3)	-0.1 (-0.4;0.4)	0.1 (-0.5;0.7)	0.630	0.557
17:1 n-9	0.1 (0.1;0.1)	0.0 (0;0)	0.0 (0;0)	0.1 (0.1;0.2)	0.0 (-0.1;0)	0.0 (0;0)	0.655	0.419
18:1 n-9	19 (18;21)	0.2 (-1.3;1.2)	**1.3 (0.2;2.4)** **	20 (18;21)	-0.5 (-1.6;0.5)	0.8 (-1.5;1.7)	0.317	0.072
18:1 n-11	2.1 (2;2.4)	0 (-0.1;0.2)	**0.2 (0;0.3)*** **	2.3 (2.1;2.3)	-0.1 (-0.2;0.1)	0.1 (-0.2;0.2)	0.240	**0.033**
22:1 n-9	0.1 (0.1;0.2)	-0.1 (-0.1;0.1)	**-0.1 (-0.2;-0.1)*** **	0.1 (0.1;0.2)	-0.1 (-0.1;0.1)	**-0.1 (-0.2;0)*** **	0.983	0.419
18:2 n-6	30 (26;32)	**5.2 (3.4;7.2)*** **	0.4 (-1.7;2.4)	28 (26;30)[#]	**6.7 (4;8.4)*** **	2.0 (-0.7;4.4)	0.151	0.158
18:3 n-3	0.4 (0.4;0.7)	0.1 (-0.1;0.2)	0.1 (-0.1;0.2)	0.4 (0.4;0.7)	0.0 (-0.1;0.1)	0.0 (-0.1;0.1)	0.547	0.226
18:3 n-6	0.4 (0.3;0.5)	0.0 (-0.1;0.2)	0.0 (-0.1;0.1)	0.3 (0.3;0.5)	0.0 (-0.1;0.1)	0.0 (-0.1;0.1)	0.326	0.960
20:2 n-6	0.1 (0.1;0.1)	**-0.1 (-0.1;0)** **	0.0 (0;0)	0.1 (0.1;0.2)	**-0.1 (-0.1;0)*** **	0.0 (0;0)	0.570	1.000
20:2 n-9	0.2 (0.1;0.2)	0.0 (0;0)	**-0.1 (-0.1;0)** **	0.3 (0.1;0.2)	0.0 (0;0)	0.0 (0;0)	0.630	0.164
20:3 n-6	1.6 (1.4;1.8)	0.0 (-0.1;0.4)	-0.1 (-0.3;0.1)	1.7 (1.4;1.8)	**0.2 (-0.1;0.5)* **	0.0 (-0.3;0.2)	0.438	0.431

Suppl. Table 2 (continued)	Intervention group			Control group			Intervention vs. control groups (p)	
	**BL (n = 33)**	**Δ V1-BL (n = 26-33)**	**Δ V2-BL (n = 23-29)**	**BL (n = 35)**	**Δ V1-BL (n = 29-35)**	**Δ V2-BL (n = 28-32)**	**Δ V1-BL**	**Δ V2-BL**
**Fatty acids [%]**								
20:3 n-9	0.1 (0.1;0.1)	0.0 (0;0)	0.0 (0;0.1)	0.1 (0.1;0.1)	0.0 (0;0)	0.0 (0;0.1)	0.408	0.698
20:4 n-6	7.2 (5.9;8)	**-2.6 (-3.3;-1.8)*** **	**-0.6 (-1.3;0.1)** **	7.3 (6.4;7.8)	**-2.9 (-3.5;-2.2)*** **	-0.2 (-0.9;0.6)	0.262	0.082
20:5 n-3	0.9 (0.4;1.1)	**0.3 (0;0.5)** **	0.1 (-0.1;0.3)	1.1 (0.5;1.2)	0.0 (-0.2;0.4)	-0.1 (-0.3;0.3)	**0.049**	0.479
22:5 n-3	0.6 (0.5;0.6)	**-0.5 (-0.6;-0.4)*** **	**0.1 (0;0.2)* **	0.6 (0.5;0.8)	**-0.5 (-0.7;-0.3)*** **	**0.1 (-0.1;0.3)** **	0.705	0.683
22:6 n-3	2.5 (1.9;3)	**1.5 (0.9;2.5)*** **	**0.3 (-0.1;0.9)* **	2.6 (2;3.5)	**0.9 (0.5;1.6)*** **	0.0 (-0.6;0.4)	0.071	**0.014**
Total fatty acids	100			100				
Total SFAs	32 (30;34)	**-3.3 (-5.5;-2.2)*** **	**-1.9 (-3.5;-0.7)*** **	32 (31;33)	**-4.1 (-4.8;-2.2)*** **	**-1.1 (-2.5;0.1)* **	0.513	0.152
Total MUFAs	24 (23;27)	-0.4 (-2.4;1.7)	**1.6 (-0.1;3.3)** **	25 (24;27)	-0.7 (-1.8;0.7)	0.4 (-1.3;1.9)	0.655	0.072
Total PUFAs	44 (41;47)	**3.8 (2.4;8.4)*** **	0.1 (-2.2;2.6)	43 (41;45)	**4.9 (1.3;6.8)*** **	1.3 (-2.1;4.1)	0.823	0.364
Total n-3	4.0 (3.5;5.8)	**1.4 (0.5;2.3)*** **	**0.8 (-0.1;1.4)* **	4.3 (3.5;5.8)	0.5 (-0.3;1.4)	-0.1 (-1.1;0.9)	**0.027**	**0.039**
Total n-6	39 (36;42)	**2.8 (1;4.3)*** **	-0.6 (-2.2;1.3)	37 (36;40)	**4.2 (1.4;5.5)*** **	2.1 (-1.6;4.5)	0.269	0.115
Total n-9	19 (19;22)	0.6 (-1.4;1)	**1.1 (0.1;2.4)** **	21 (19;21)	-0.4 (-1.8;0.2)	0.4 (-1.4;1.5)	0.352	0.086
n-3 / n-6	0.1 (0.1;0.1)	**0.1 (0;0.1)** **	**0.1 (0;0.1)* **	0.1 (0.1;0.2)	0.0 (0;0)	0.0 (0;0)	**0.029**	**0.018**
n-6 / n-3	9.6 (7.3;11.2)	**-1.5 (-3.2;0)** **	-1.3 (-3;0.4)	8.8 (6.2;10)	-0.1 (-1.8;1.7)	0.5 (-1.5;2.3)	**0.008**	**0.013**
Omega-3-Index[3]	2.8 (2.4;4.2)	**1.5 (1.1;2.5)*** **	**0.5 (-0.1;1.3)* **	3.1 (2.6;4.6)	**1.0 (0.4;1.9)** **	-0.2 (-0.8;0.5)	**0.044**	**0.045**
**Biomarker**								
LBP [µg/ml]	3.4 (3.1;3.8)	**-0.3 (-0.6;0.1)** **	**-0.3 (-1.1;-0.1)*** **	3.5 (3.1;4)	**-0.2 (-0.8;-0.1)*** **	0.0 (-0.6;0.3)	0.212	**0.017**
Zonulin [ng/mg]	170 (132;328)	**-76 (-164;-12)** **	**-74 (-197;15)** **	180 (60;264)	**-59 (-186;15)* **	-10 (-117;24)	0.583	0.175

[1]Eicosanoic acid, [2]Phytanic acid, [3]eicosapentaenoic acid (20:5) + docosahexaenoic acid (22:6). Median and interquartile ranges (25th;75th percentiles) are shown. Difference between the study groups at baseline and difference between the group's shifts was tested using the Mann-Whitney *U* test ([#]difference between the groups at baseline; p < 0.05). Within group difference between baseline and V1/V2 was tested using the Wilcoxon signed-rank test (*p < 0.05; **p < 0.01; ***p < 0.001). Further abbreviations: a; anteiso fatty acid; i, iso fatty acid; MUFAs, monounsaturated fatty acids; n-, Omega- (fatty acid); PUFAs, polyunsaturated fatty acids; SFAs, saturated fatty acids.

Supplementary Table 3. Multiple linear regression models showing the effect of fecal short-chain fatty acids, as well as potential confounders on intestinal barrier integrity. Shown are the data for the shift between baseline (BL) and month 3 (Δ V1-BL), and for the shift between BL and month 12 (Δ V2-BL). There were no significant results in the control group for Δ V2-BL.

		**Intervention group**		**Control group**
		**Δ V1-BL**	**Δ V2-BL**	**Δ V1-BL**
**Outcome**	**Predictor**	**ß / R^2 / n[a]**	**ß / R^2 / n[a]**	**ß / R^2 / n[a]**
Δ LBP [µg/ml]	SFA [% plasma]	0.138*		
	VT1 [VO_{2max}]	0.006		
	BMI [kg/m^2]	0.009		
	Diseased[2] [yes/no]	-0.250		
	R^2 / n	0.45 / 20		
Δ LBP [µg/ml]	DHA [% plasma]	**-0.187#**		**-0.109***
	VT1 [VO_{2max}]	0.013		0.0014
	BMI [kg/m^2]	-0.040		0.316#
	Diseased[2] [yes/no]	-0.185		-0.570#
	R^2 / n	0.42 / 18		0.37 / 24
Δ LBP [µg/ml]	Propionate [mg/g]	**-0.057***	**-0.075***	
	VT1 [VO_{2max}]	0.010	0.005	
	BMI [kg/m^2]	0.025	0.163	
	Diseased[2] [yes/no]	-0.192	-0.164	
	R^2 / n	0.64 / 18	0.68 / 16	
Δ LBP [µg/ml]	Butyrate [mg/g]	**-0.064****	**-0.057***	
	VT1 [VO_{2max}]	0.011	0.005	
	BMI [kg/m^2]	0.012	0.256	
	Diseased[2] [yes/no]	-0.263	0.132	
	R^2 / n	0.64 / 18	0.60 / 16	
Δ Zonulin [ng/mg]	DHA [% plasma]	**-58.0***	**-108.0***	
	VT1 [VO_{2max}]	3.585	-1.224	
	BMI [kg/m^2]	-62.06	-17.14	
	Diseased[2] [yes/no]	1.529	-40.04	
	R^2 / n	0.40 / 18	0.42 / 15	
Δ Zonulin [ng/mg]	Propionate [mg/g]	**-21.8****		**-10.4***
	VT1 [VO_{2max}]	1.93		-0.91
	BMI [kg/m^2]	-40.6		-41.6
	Diseased[2] [yes/no]	-8.7		-134.4
	R^2 / n	0.71 / 19		0.65 / 16
Δ Zonulin [ng/mg]	Butyrate [mg/g]	**-20.7****		**-21.1#**
	VT1 [VO_{2max}]	2.86		2.50
	BMI [kg/m^2]	-48.1		30.1
	Diseased[2] [yes/no]	-32.9		106.9
	R^2 / n	0.70 / 19		0.62 / 24

Statistics: Multiple linear regressions (#p ≤ 0.070; *p < 0.050; **p < 0.010). [a]Number of analyzed cases (individuals without missing data in the outcome and predictors). [2]Previously diagnosed with breast cancer. Only results with p ≤ 0.07 are shown. Highlighted results were found (i) consistently for the intervention group and the control group and/or (ii) consistently for the two shifts ΔV1-BL and ΔV2-BL. Further abbreviations: BMI, body mass index; DHA, docosahexaenoic acid; LBP, lipopolysaccharide binding protein; SFA, saturated fatty acids; VT1, ventilatory threshold 1.
